# Chimeric Vaccine Stimulation of Human Dendritic Cell Indoleamine 2, 3-Dioxygenase Occurs via the Non-Canonical NF-κB Pathway

**DOI:** 10.1371/journal.pone.0147509

**Published:** 2016-02-16

**Authors:** Nan-Sun Kim, Jacques C. Mbongue, Dequina A. Nicholas, Grace E. Esebanmen, Juli J. Unternaehrer, Anthony F. Firek, William H. R. Langridge

**Affiliations:** 1 Center for Health Disparities and Molecular Medicine, Department of Basic Sciences, Loma Linda University School of Medicine, Loma Linda, California, United States of America; 2 Department of Molecular Biology, Chonbuk National University, Jeon-Ju, Republic of Korea; 3 Loma Linda University School of Medicine, Department of Basic Sciences, Division of Physiology, Loma Linda, California, United States of America; 4 Loma Linda University School of Medicine, Department of Earth and Biological Sciences, Loma Linda, California, United States of America; 5 Loma Linda University School of Medicine, Department of Basic Sciences, Division of Biochemistry, Loma Linda, California, United States of America; 6 Endocrinology Section, JL Pettis Memorial VA Medical Center, Loma Linda, California, United States of America; Istituto Superiore di Sanità, ITALY

## Abstract

A chimeric protein vaccine composed of the cholera toxin B subunit fused to proinsulin (CTB-INS) was shown to suppress type 1 diabetes onset in NOD mice and upregulate biosynthesis of the tryptophan catabolic enzyme indoleamine 2, 3-dioxygenase (IDO1) in human dendritic cells (DCs). Here we demonstrate siRNA inhibition of the NF-κB-inducing kinase (NIK) suppresses vaccine-induced IDO1 biosynthesis as well as IKKα phosphorylation. Chromatin immunoprecipitation (ChIP) analysis of CTB-INS inoculated DCs showed that RelB bound to NF-κB consensus sequences in the IDO1 promoter, suggesting vaccine stimulation of the non-canonical NF-κB pathway activates IDO1 expression *in vivo*. The addition of Tumor Necrosis Factor Associated Factors (TRAF) TRAF 2, 3 and TRAF6 blocking peptides to vaccine inoculated DCs was shown to inhibit IDO1 biosynthesis. This experimental outcome suggests vaccine activation of the TNFR super-family receptor pathway leads to upregulation of IDO1 biosynthesis in CTB-INS inoculated dendritic cells. Together, our experimental data suggest the CTB-INS vaccine uses a TNFR-dependent signaling pathway of the non-canonical NF-κB signaling pathway resulting in suppression of dendritic cell mediated type 1 diabetes autoimmunity.

## Introduction

Type 1 diabetes (T1D) is a well-studied prototypic tissue specific autoimmune disease resulting from auto-reactive lymphocyte destruction of the pancreatic islet insulin-producing β-cells [1,2]. The progressive loss of islet β-cell function leads to insulin deficiency and high blood glucose levels (hyperglycemia). Increased levels of cellular oxidative stress and chronic inflammation generated by hyperglycemia leads to neural and circulatory complications that result in an early mortality from amputation, loss of kidney function, blindness, heart attack, and stroke [3,4].

Due to the high cost and extended duration of palliative patient care, there is an urgent need for therapeutics that can safely deliver specific effective therapy that protects against the onset and reverses the progression of tissue specific autoimmunity. Dendritic cells, which are considered the most prominent subset of professional antigen presenting cells (APC), have been implicated in the initiation of diabetes related islet β-cell destruction [5–7]. Effective immunological suppression strategies include chimeric vaccines that link immuno-stimulatory molecules (adjuvants) with autoantigens to enhance vaccine efficacy [8–10]. Prominent among the adjuvants used is the cholera toxin B-subunit (CTB) [11]. C-terminal linkage of CTB to the diabetes autoantigen proinsulin (CTB-INS), generated a fusion protein shown to protect against T1D onset [10]. Oral immunization experiments showed that feeding small amounts (2–20μg) of CTB-INS vaccine protein alone or in recombinant plant tissues effectively suppressed β-cell destruction and clinical diabetes in adult non-obese diabetic (NOD) mice [8,9,12].

Proteomic analysis of human dendritic cells inoculated with CTB-INS revealed strong up-regulation of the tryptophan catabolic enzyme indoleamine 2, 3-dioxygenase (IDO1) [13]. Previous observations showed that CD40 ligand (CD40L) induced IDO1 biosynthesis in human DCs through activation of the non-canonical NF-κB signaling pathway [14]. Thus, we assessed the requirement for NF-κB activation in vaccine up-regulation of IDO1 using ACHP and DHMEQ, pharmacological inhibitors of NF-κB [13]. While these experiments revealed vaccine stimulation of NF-κB was essential to activate IDO1 biosynthesis, the relative contributions of canonical and non-canonical NF-κB pathways required for CTB-INS induction of IDO1 biosynthesis remained undetermined.

The NF-κB family in mammals is composed of five members, including c-Rel, RelA also known as p65, NF-κB1 known as p50, RelB, and NF-κB2 known as p52. These NF-κB family members form a variety of dimeric complexes capable of trans-activating numerous target genes through binding to the κB enhancer [15]. The NF-κB subunit proteins are normally found inactive in the cell cytoplasm due to binding by a family of inhibitors that include IκBa and several additional related ankyrin repeat-containing proteins [15,16].

Due to the diversity of NF-κB functions, its activity is under tight control at multiple levels by positive and negative regulatory elements. Under resting conditions, NF-κB dimers are bound to inhibitory IκB proteins that retain NF-κB complexes in the cytoplasm. In the canonical signaling pathway, the degradation of IκB inhibitor proteins is initiated through stimulus-induced phosphorylation by IκB kinase (IKK), a molecular complex consisting of two catalytically active kinases, IKKα and IKKβ, and their regulatory subunit IKKγ (NEMO). Phosphorylation of IκB proteins target them for ubiquitination and proteasome degradation, releasing the NF-κB RelA (p65) and p50 protein dimers for translocation into the nucleus.

The non-canonical NF-κB signaling pathway was discovered during analysis of non-canonical p100 subunit processing [15,17]. In addition to serving as a precursor of the functional p52 subunit, p100 was shown to function like an IκB inhibitor molecule, preferentially inhibiting RelB nuclear translocation [15,18]. Partial proteasome processing of p100 serves to generate p52 and to induce nuclear translocation of the RelB/p52 heterodimer as RelB binds to DNA with p52. The p52 subunit is actively generated predominantly in specific immune cell types including B-cells and dendritic cells, leading to the idea that p100 processing might be a signal-regulated event. Indeed the NF-κB-inducing kinase (NIK) is required for p100 processing and is required for *in vivo* p100 processing in splenocytes [17,19]. The first component of the non-canonical signaling pathway to be identified was NIK, a MAP-kinase kinase kinase (MAP3K) member originally implicated in NF-κB activation by the TNF receptor (TNFR) pathway [20]. To date, all of the non-canonical NF-κB inducers identified are known to signal through NIK [14,21,22].

Here we focus on identification of non-canonical NF-κB signaling pathway contributions to CTB-INS vaccine induction of IDO1 in human dendritic cells as a prerequisite for application of chimeric vaccine immune suppression strategies in the clinic.

## Materials and Methods

### Construction of a bacterial expression vector containing the cholera toxin B subunit–proinsulin gene

**Fig 1:**
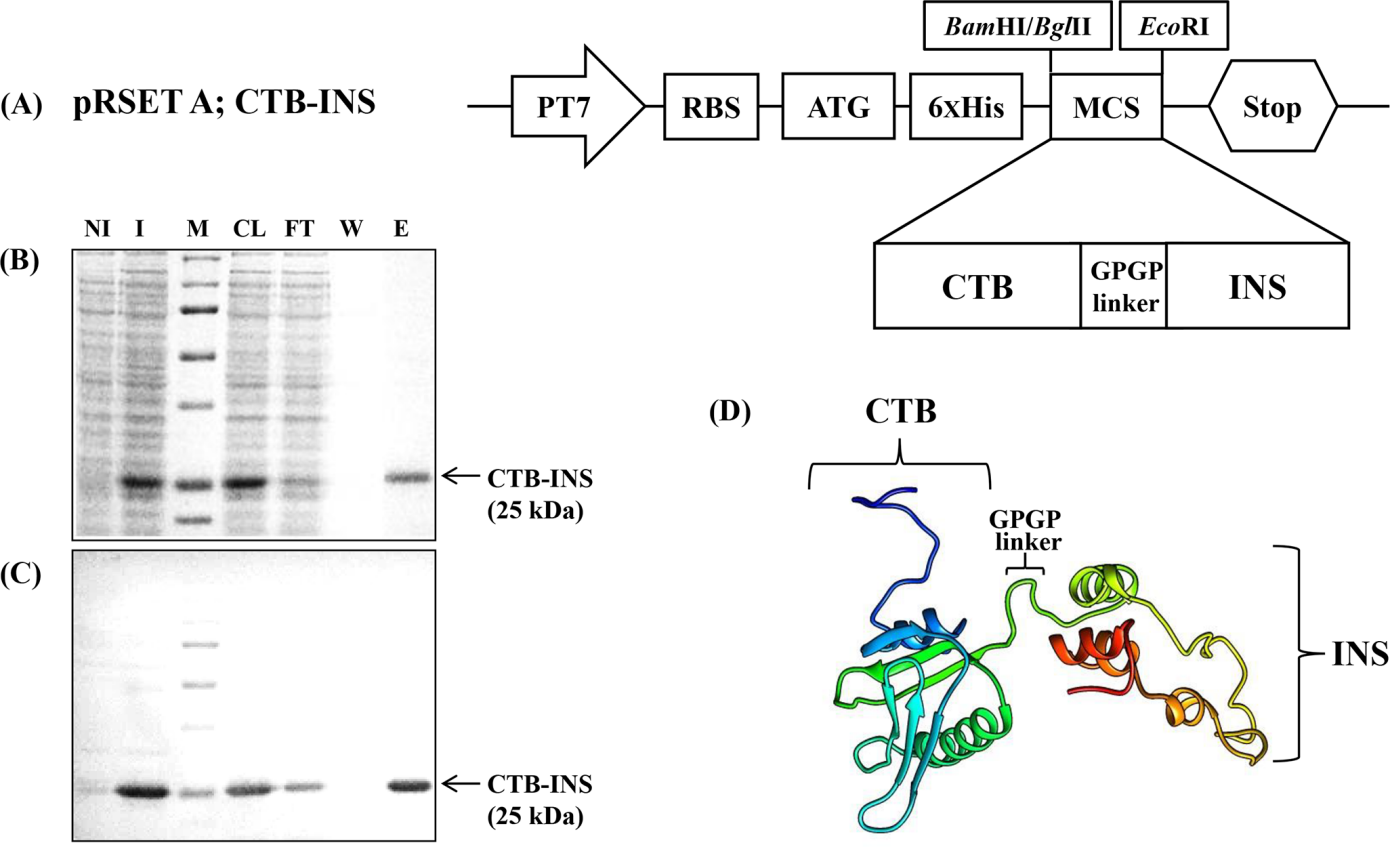
CTB-INS fusion protein was expressed from the *E.co*li pRSET A expression vector and purified using Ni-NTA agarose with the indicated imidazole concentration in the wash and elution steps. Panel **(A)** is a plasmid map of the *E*. *coli* expression vector pRSET A (Invitrogen, Carlsbad, CA), carrying the CTB-INS fusion gene. Panel **(B)** show the SDS-PAGE. Proteins were visualized by Coomassie staining. Lane NI: non-induced *E*. *coli* (BL21) cell; I: induced E. *coli* cell; M: protein size marker; CL: cell lysate; FT: flow-through; W: wash; E: elution. Panel **(C)** Western blot detection of recombinant CTB-INS fusion protein identified with anti-CTB primary antibody. The arrow indicates the purified CTB-INS proteins. Panel **(D)** shows the predicted CTB-INS protein structure using its protein sequence generated by the RaptorX server.

A DNA sequence encoding 258bp of the human proinsulin gene (INS M12913.1) was linked to the carboxyl-terminus of a DNA fragment (309bp) encoding the cholera toxin B subunit gene (CTB U25679.1) to generate the fusion gene CTB-INS according to a previously used protocol [13](**Fig 1**).

### Expression and purification of CTB-INS fusion protein in *E*. *coli*

The *E*. *coli* strain BL21 was transformed with pRSET-CTB-INS as previously described [13].

### Ethics

Ex vivo experiments on monocyte-derived DCs were performed, with aphaeresis blood provided by the Life Stream Blood Bank (San Bernardino, CA). These experiments were approved by the *Loma Linda University Adventist Health Sciences Center Institutional Review Board* and blood donor written consent. Blood donor information was anonymized and de-identified prior to the study

### Isolation and culture of monocyte—derived dendritic cells from human peripheral blood

Monocyte-derived dendritic cells (MoDCs) were prepared from freshly collected human peripheral blood cells isolated from aphaeresis filter cones obtained from the LifeStream blood bank (San Bernardino, CA). The blood was incubated with a red blood cell lysis buffer (3.0 mL Lysis Buffer/ mL of blood) containing 8.3g/L NH_4_Cl, 1g/L KHCO_3_, and 1.8 mL 5% EDTA (Boston Bioproducts), and centrifuged for 5 minutes at 1,500 rpm at 4°C in a Beckman Coulter Allegra X-15R centrifuge, equipped with a SX4750 rotor. After a total of 3 washes in PBS to remove cellular debris and hemoglobin CD14^+^ monocytes were obtained from the total lymphocyte fraction by incubation with anti-CD14 antibodies bound to magnetic beads for 15 minutes at 4°C (Miltenyi Biotech, Auburn, CA). The monocytes were separated from other immune cells by binding to a magnetic MACS column followed by elution of all other leucocytes (Miltenyi Biotech, Auburn, CA). The monocytes were eluted from the column and cultured at a concentration of 2–9 x 10^6^ cells/well in 6-well non-pyrogenic polystyrene culture plates in RPMI 1640 culture medium (Mediatech Inc. Manassas, VA, USA), supplemented with 10% FBS, 1 mM glutamine, 100 U/ml penicillin, 100 μg/ml streptomycin, 50 ng/ml human recombinant GMCSF, and 10 ng/ml human recombinant IL-4 (ProSpec-Tany), at 37°C in a humidified atmosphere of 5% CO_2_ (Preprotech, Rocky Hill, NJ). The monocyte cell culture was fed at 2-day intervals by gentle replacement of 50% of the medium with fresh pre-warmed culture medium. The cells were cultured for a total of 6 days to allow monocyte differentiation into DCs prior to vaccine treatment. The cells were monitored by phase contrast microscopy to assess dendrite formation, a marker indicating DC differentiation.

### IDO1 protein synthesis in vaccinated dendritic cells

Approximately 2–9 x 10^6^ monocyte-derived DCs generated from each of several subjects were inoculated with CTB-INS (0.1, 0.5, 1.0, 2.5, 5.0 and 10 μg/ml), 500 ng/ml of CD40L (Immunex, Seattle, WA), 500 ng/ml of TRAF 2,3 binding peptide (Proteintech Group, San Diego, CA) and 500 ng/ml of TRAF 6 binding peptide (Proteintech Group). The vaccinated DCs were incubated for 6, 12, 24, 48 or 96 hours and lysed in buffer C (20 mM HEPES, 0.42 M KCl, 26% Glycerol, 0.1 mM EDTA, 5 mM MgCl_2,_ 0.2% NP40, 37°C) containing a tablet of complete protease inhibitor (Roche, Basel, Switzerland) according to the manufacturer instructions. At least 50 μg of protein isolated from the total DC lysate was separated by electrophoresis on a 12% polyacrylamide gel (SDS-PAGE). After transfer of the separated proteins to polyvinylidene difluoride (PVDF) membranes (Millipore, Temecula, CA), the presence of IDO1 protein (NP_002155.1) was detected by incubation of the blot for 12 hours at 4°C with an anti-IDO1 rabbit monoclonal primary antibody (Cat. 04–1056, clone EPR1230Y) (Millipore, Temecula, CA). For signal detection, the blot was washed 3 times with PBST (1X PBS, 0.02% tween 20, pH 7.4) and incubated for 2 hours at room temperature in the presence of a monoclonal anti-rabbit IgG γ-chain specific alkaline phosphatase conjugated secondary antibody (Cat. A-2556, clone RG-96) (Sigma-Aldrich). The immunoblots were washed 3 times in PBST and incubated in 200 μL of Novex® AP chemiluminescent substrate (Invitrogen™) for 5 minutes prior to exposure to x-ray film (Kodak X-Omat) for 3 minutes. The IDO signal intensity was quantified via Image J software v. 1.48h. (Image J, NIH).

### Small interfering RNA (siRNA) transfection

No pharmacological inhibitors for IKKα exist that selectively block the non-canonical pathway of NF-κB activation [14,23]. Here we used siRNA to specifically target this pathway. To define the regulatory effect of the NF-κB pathway on CTB-INS-induced IDO expression, human NIK-small interfering RNA (NIK siRNA, sc-36065) and non-targeting siRNA (Control siRNA Fluorescein conjugate, sc-36869) were purchased from Santa Cruz Biotechnology (Santa Cruz, Delaware, CA, USA). Monocytes were cultured in six-well plates for 4 days with 50 ng/ml of hGM-CSF and 10 ng/ml of IL-4 for differentiate the mature DCs. siRNAs were transfected into DC cells using Lipofectamine® RNAiMAX reagent (Invitrogen, Carlsbad, CA, USA) according to the manufacturer’s protocol. Three microliters of 10 μM siRNA was mixture in 150 μl of Opti-MEN (Gibco-Life Technologies, Paisley,UK), while 9 μl of Lipofectamine® RNAiMAX reagent was incubated in 150 μl of Opti-MEN at room temperature for 5 min. Then the diluted siRNA and Lipofectamine® RNAiMAX reagent were incubated for a further 20 min at room temperature for complex formation. The complexes were added to wells. The final siRNA concentration was 25 pmol. DC continued to be incubated at 37°C in 5% humidified CO_2_ for 48 h which was sufficient to significantly knock down the target protein levels. Expression of IDO1 was induced by 5 μg of CTB-INS for 24h after siRNA transfection. To evaluate transfection efficiency, FITC-labeled control RNA was substituted for siRNA. After 24 hours, incubation, the transfected DCs were analyzed by fluorescence microscopy for intracellular FITC content. To confirm IKKα phosphorylation, DCs were treated with 10μg/ml of CTB-INS for 6hrs after transfection and lysed in 100 μL buffer C/well containing phosphatase inhibitors (50 mM Sodium-beta-glycerophosphate, 1mM Sodium fluoride, 1 mM Sodium-ortho-vanadate). Western blot analysis with Anti-IKK alpha (phospho S176+S180) (Abcam 1:1000) and anti-rabbit IgG whole molecule conjugated AP (Sigma-Aldrich 1:1000) was performed prior to band detection on x-ray film (Kodak X-Omat) of IKKα.expression.

### Total RNA preparation and reverse transcription polymerase chain reaction (RT-PCR)

Total RNA from 1 Х 10^6^ DC was prepared using Trizol (Invitrogen, Carlsbad, CA, USA) and complementary DNA was synthesized from 2 μg total RNA with oligo (dT) primer in a 20 μl reaction volumn according to the manufacturer’s recommendations (Thermo Fisher Scientific Inc, Waltham, MA, USA). Polymerase chain reaction (PCR) amplification was performed at 95°C for 1m, 58°C for 1m, 72°C for 30s, and PCR was done for 35 cycles. The primers used in this study were NIK (h)-PR (sc-36065-PR, Santa Cruze), product size 537 bp; and β-actin forward, 5’-GCA TTG CTT TCG TGT AAA TTA TGT-3’ β-actin reverse, 5’-ACC AAA AGC CTT CAT ACA TCT CA-3’, product size 211 bps. The PCR products were size-separated on 1.5% agarose gels and visualized by Et-Br DNA gel staining.

### ChIP analysis of CTB-INS induction of NF-κB activation *in vivo*

A chromatin immunoprecipitation (ChIP) assay was performed to identify the specific binding sequences in the IDO1 promoter region for the non-canonical NF-κB subunits RelB protein using a MAGnify^TM^ Chromatin Immunoprecipitation System (Invitrogen) according to the manufacturer’s instructions. 3–8 x 10^6^ Human dendritic cells were left unstimulated or were stimulated with CTB-INS for 3h, after which DC cells were harvested and washed with 1ХPBS and were fixed in formaldehyde (Sigma, St Louis, MO, USA) to a final concentration of 1%. After 10 min, 1.25M glycine was added to stop crosslinking reaction. After centrifugation, cells were lysed for 5 min in Lysis buffer supplemented with protease inhibitors. Chromatin was sheared by sonication (5 Х 12s at one-fifth of the maximum potency) with a Sonic 60 Dismembrator (Fisher Scientific, Sunnyvale, CA, USA), centrifuged to pellet debris, and diluted in Dilution Buffer which was recommend in manufacturer’s instructions. Fragmented chromatin was immunopreciated with a ChIP-grade antibody against RelB (GeneTex, Irvine, CA, USA) which was coupled with Dynabeads ®, at 4°C overnight. Immune complexes were washed with IP Buffer 1 and 2 in the DynaMag^TM^-PCR Magnet. For reversing the crosslinking, Reverse Crosslinking Buffer with proteinase K was added both input control (fragmented chromatin without immunoprecipitation) and immune complexes and incubate at 55°C for 15 min. The DNA was purified with DNA Purification Magnetic Beads and buffers provided in the kit according to the manufacturer’s instructions. The immunoprecipitated DNA was used in each real-time PCR assay using primers specific for indicated regions of the DNA. The primers were designed using Primer Express 2.0 software (PE Applied Biosystems, USA) under default parameters. The primers that were used are as follows: 5’-CGT TAA TGG TGA ATT CAG TGA TG-3’ (2732 F1) and: 5’-TGC AGA GGG ACC TTC ATT CAA G-3’ (2732 R1), 5’-GGT AGA GAT GTT CCT CAG GCA G-3’ (2961 F2) and 5’-CTC TAT GGC CTC CTA CAT CTG-3’ (2961 R2), 5’-TGA GTT CTG GCT TTC AGG AG-3’ (3072 F3) and 5’-GAT CTT GTC TTC ATT CAC CTT G-3’ (3072 R3). Real-time PCR amplification reactions were performed using SYBR Green detection chemistry and run as triplicate samples on 96-well plates using the CFX 96^TM^ Real-Time PCR Detection System (Bio-Rad). The PCR reactions were prepared in a total volume of 25 μl containing: 5 μl of Chip or input template DNA, 2 μl of each amplification primer (final concentration 50 nM), and 12.5 μl of 2Х iQ SYBR Green Supermix (Bio-Rad). The cycling conditions were set as follows: an initial denaturation step of 95°C for 10 min to activate the iTaq DNA polymerase, followed by 40 cycles of denaturation at 95°C for 15 s, and annealing at 60°C for 1 min. The amplification process was followed by a melting curve analysis, ranging from 65°C to 95°C, with temperature increasing at steps of 2°C every 1 min. Baseline and threshold cycles (Ct), were automatically determined using the Bio-Rad CFX Manager 2.1. The samples were electrophoresed on a 1.5% (w/v) agarose gel, and the banding pattern observed under UV light. Two biological replicates for each sample were used for real-time PCR analysis and three technical replicates were analyzed for each biological replicate.

### Blocking TNFR activation of IDO1 biosynthesis

Peptides containing the CD40 receptor TRAF2, 3 and TRAF6 binding sites were linked to the TAT_47–57_ cell penetrating peptide. The sequences for the CD40–TRAF2, 3 and the CD40–TRAF6 blocking peptides were NH_2_-NTAAPVQETLHGYGRKKRRQRRR-OH and NH_2_-KQEPQEI*D*FPDD YGRKKRRQRRR-OH respectively. The TAT_47–57_ sequence is underlined. Control peptides consisted of either TAT_47–57_ alone or TAT_47–57_ linked to a scrambled peptide. The peptides were manufactured by Proteintech Group (San Diego, CA) and were low in endotoxin and > 98% pure as measured by HPLC [24,25].

### CTB-INS amino acid sequence alignment with TNF receptor family members

The protein amino acid sequence of CTB-INS (FASTA sequence-MIKLKFGVFFTVLLSSAYAHGTPQNITDLCAEYHNTQIYTLNDKIFSYTESLAGKREMAIITFKNGAIFQVEVPGSQHIDSQKKAIERMKDTLRIAYLTEAKVEKLCVWNNKTPHAIAAISMANGPGPFVNQHLCGSHLVEALYLVCGERGFFYTPKTRREAEDLQVGQVELGGGPGAGSLQPLALEGSLQKRGIVEQCCTSICSLYQLENYCNSEKDEL), was aligned with the following tumor necrosis factor (TNF) superfamily member ligands: CD40L (Accession: NP_000065.1), TNFR14 (Accession: NP_003798.2), RANKL (Accession: NP_003692.1), and BAFF (Accession: NP_006564.1) using the T-Coffee server (http://tcoffee.crg.cat) [26]. The protein alignment graphics were constructed using Jalview software v1.6 [27].

## Results

### IDO1 expression following CTB-INS incubation

**Fig 2:**
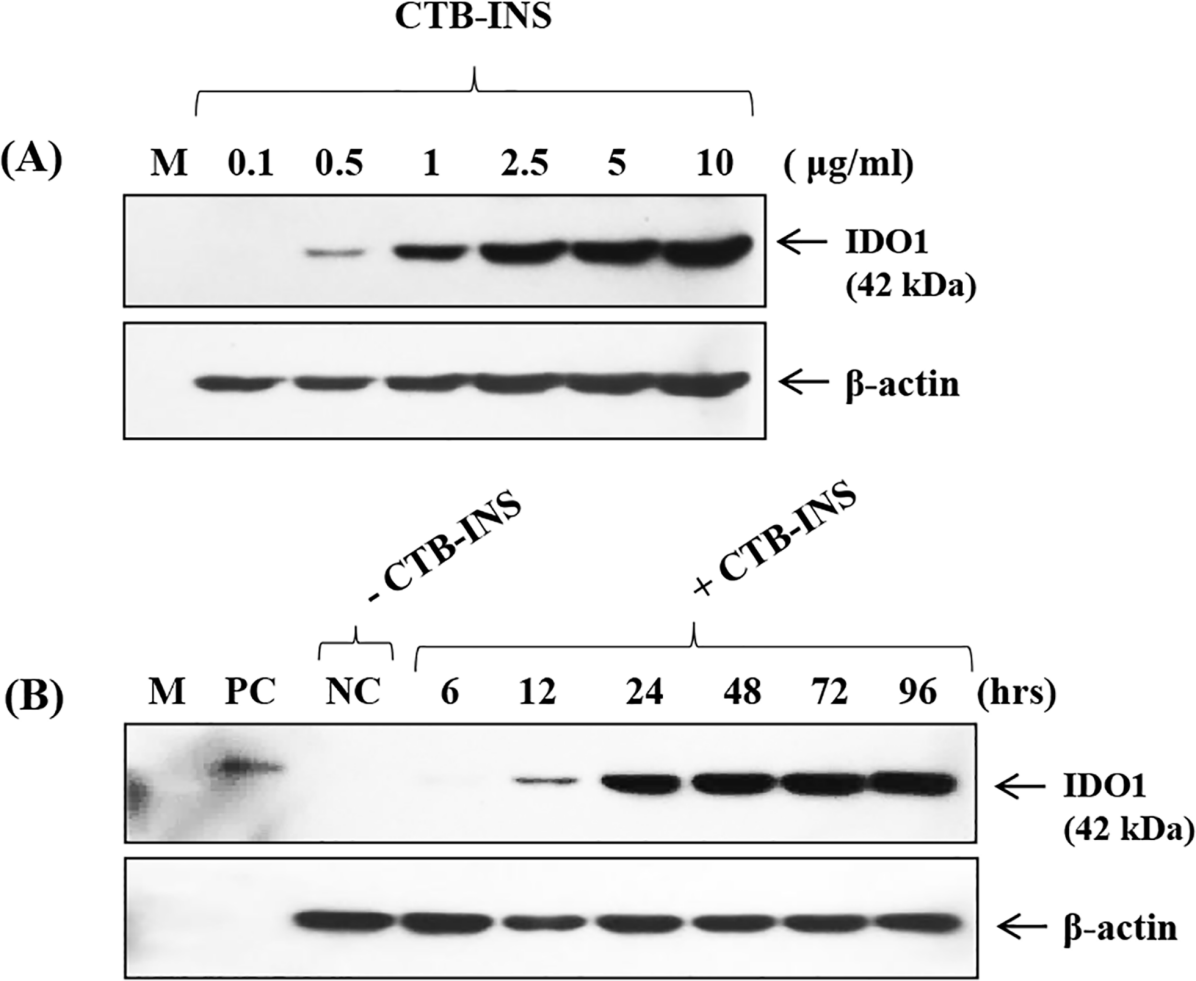
IDO1 Expression with varying concentrations of CTB-INS and incubation times and conditions. In panel **(A)** varying concentrations of CTB-INS from 0.1 to 10 μg/ml were used to assess the minimal concentration of CTB-INS needed to cause detection of IDO1 in monocyte-derived DCs. In Panel **(B)** monocyte-derived DCs were incubated with CTB-INS for 6 hrs to 96 hours with the medium changed every 2 days. IDO1 expression went increasingly unabated for 96 hours. M: Marker. **PC:** Positive Control (IDO1 recombinant protein) **NC:** Negative Control (Untreated).

Concentrations of 0.1–10 μg/ml CTB-INS were used to assess the minimal concentration of CTB-INS needed to allow detection of IDO1 in monocyte-derived DCs. Expression of IDO1 induced by CTB-INS occurred at concentrations as low as 0.5μg/ml of CTB-INS (**Fig 2A**) Monocyte-derived DCs were incubated with CTB-INS for 6 to 96 hours with the medium replaced at 2 day intervals. The levels of IDO1 in vaccine inoculated DCs increased continuously for 96 hours following vaccination **(Fig 2B).**

### CTB-INS stimulation of the non-canonical NF-κB pathway induces IDO1 synthesis in DCs

**Fig 3:**
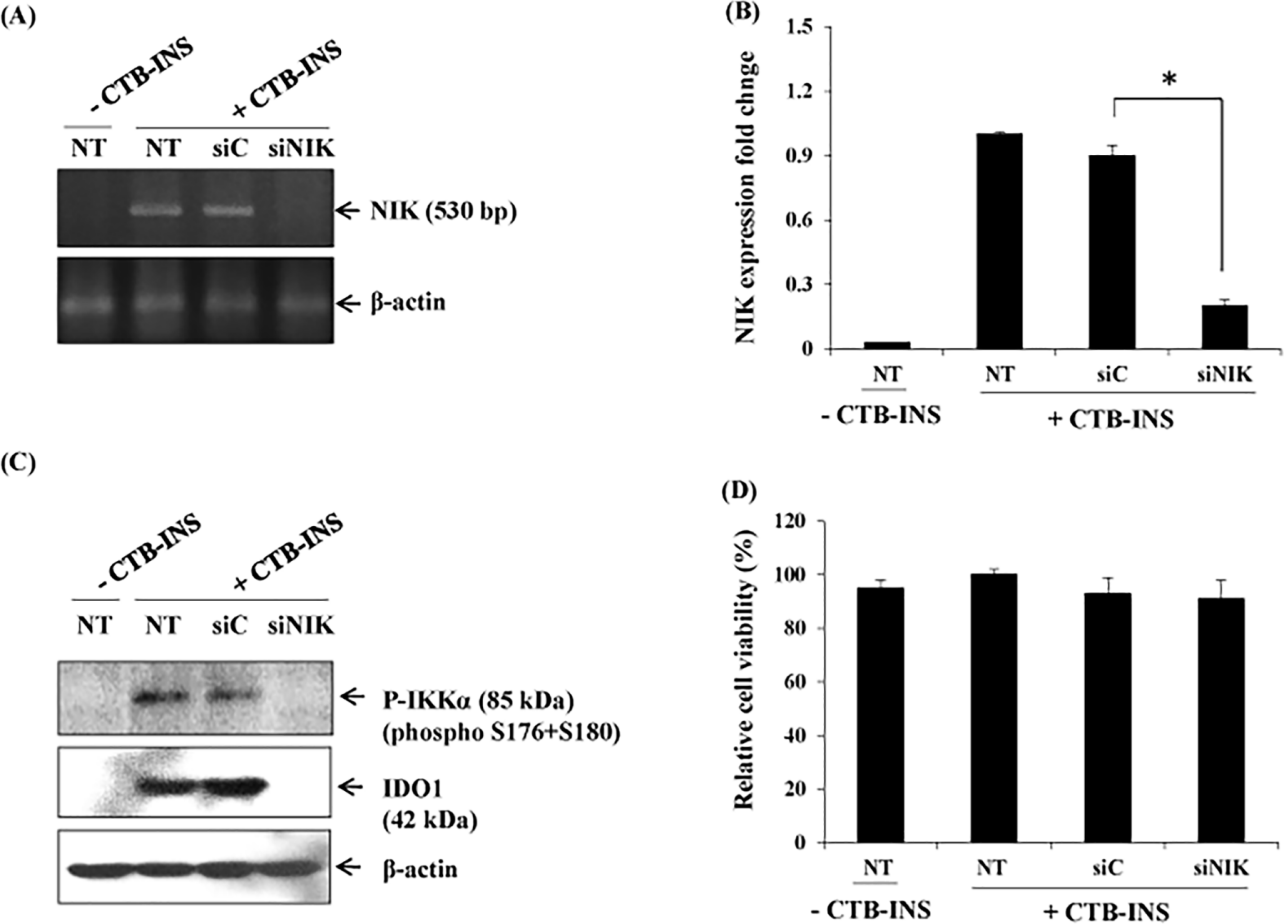
The non-canonical NF-κB pathway is required for CTB-INS-induced IDO1 expression in monocyte-derived DCs. Panel **(A)** shows NIK mRNA levels in monocyte-derived DCs transfected with NIK-specific siRNA (siNIK) or negative control (siC) examined by RT-PCR using NIK specific primers. The β-actin gene was used as an internal standard in RT-PCR. This image is representative of two independent experiments. Panel **(B)** graphic representation of NIK mRNA levels in CTB-INS vaccinated and unvaccinated DCs. Panel **(C)** show the expression of IDO1 protein and phosphorylated IKKα in mDCs transfected with NIK-specific siRNA (siNIK) or negative control (siC) examined by Western blot analysis using anti-IDO1 as the primary antibody. This image is representative of three independent experiments. Panel **(D)** shows graphic representation of the cell viability of non-transfected or transfected with NIK-specific siRNA (siNIK) or negative control (siC) in mDCs. Dendritic cell viability was measured by determination of the percentage of vaccinated DCs negative for annexin V and propidium iodide. **NT** means non-siRNA CTB-INS transfected DCs, and–or + means without CTB-proINS and with CTB-INS. Samples were assayed in triplicates and the results represent the mean ± SD of three independent experiments p = 0.002. Statistical analysis was performed using one way analysis of variance (ANOVA).

To assess non-canonical NF-κB pathway contributions to CTB-INS-induced IDO1 expression we used siRNA technology to knock down the non-canonical pathway-dependent kinase NIK and the level of IDO1 expression in treated DCs measured. The knockdown of NIK in CTB-INS stimulated DCs resulted in a significant reduction in NIK mRNA levels (**Fig 3A**) and IDO1 protein expression as well as a decrease in phosphorylated IKKα in comparison with non-specific siRNA-treated dendritic cells **(Fig 3B).** This experimental result demonstrated that CTB-INS-induced IDO expression in human DCs was dependent upon vaccine activation of the non-canonical NF-κB pathway.

### CTB-INS leads to non-canonical NF-κB RelB translocation to drive IDO1 expression in DCs *in vivo*

**Fig 4:**
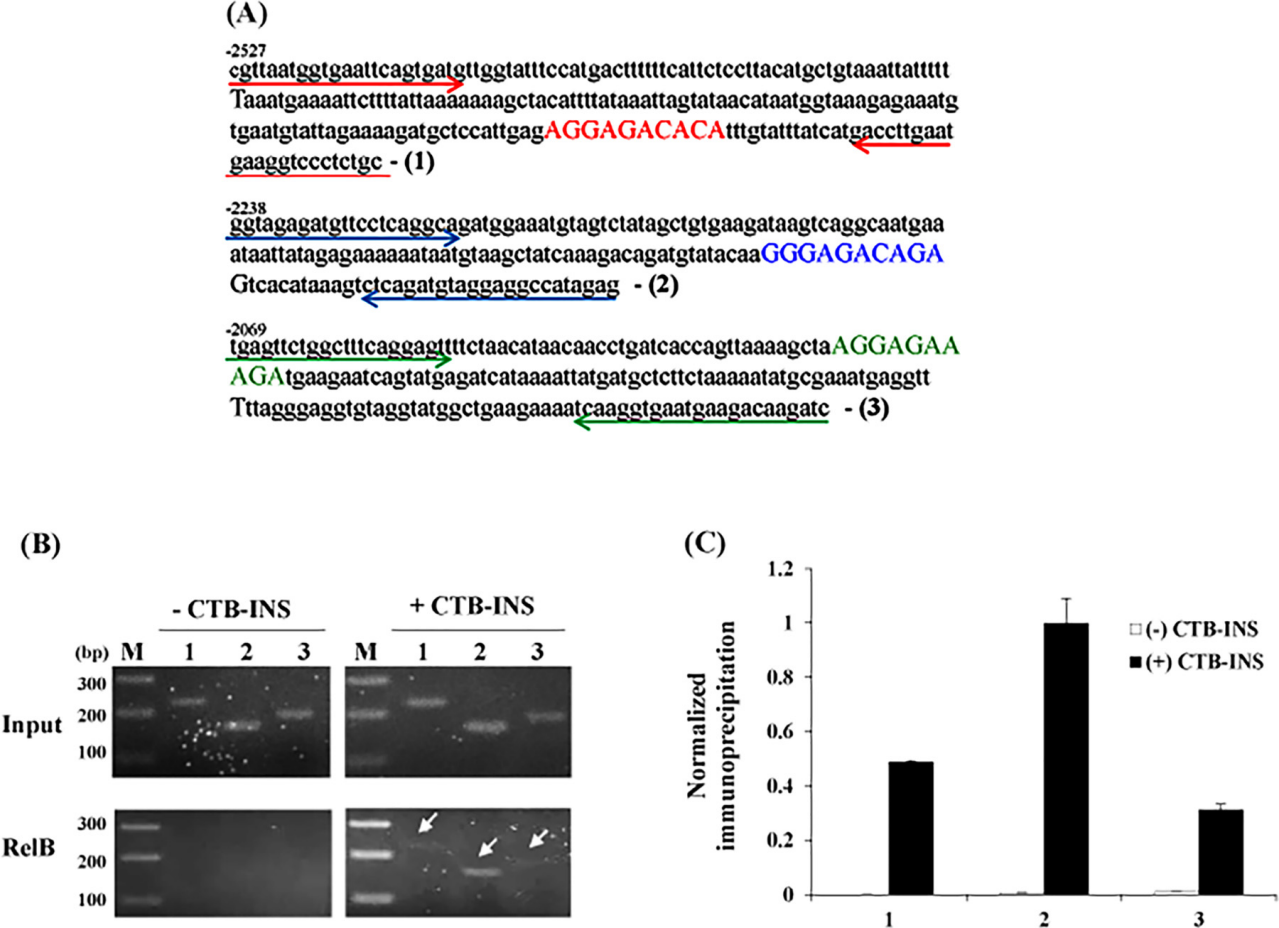
ChIP analysis showing vaccine stimulation of NF-κB RelB binding to the human dendritic cell IDO1 promoter region in vivo. Panel **(A)** shows the sequence of three partial non-canonical NF-κB RelB binding sites in the IDO1 promoter (bold nucleotides). The arrows indicate the primer sequences for detection of the three RelB binding sites. Panel **(B)** shows the PCR products after chromatin immunoprecipitation (ChIP). Immature human DCs were stimulated with the CTB-INS fusion protein vaccine for 0 (-) or 6 hr (+). Protein-DNA complexes were cross-linked, the DNA sheared and RelB genomic DNA complexes immunoprecipitated with RelB monoclonal antibody. After purification of the DNA, Real-time PCR was performed using primers flanking the three consensus NF-κB RelB binding sites in the human IDO1 promoter region shown in panel A. The Input control consists of PCR amplification of the IDO1 promoter obtained from total genomic DNA prior to immunoprecipitation. Lane M: DNA fragment size marker, Lanes 1, 2, 3: show the products of PCR amplification with primer sets detecting the three consensus RelB binding sites in the vaccinated human IDO1 promoter region (white arrows). Panel **(C)** Quantification of immunoprecipitation was performed for three experiments, by normalizing the intensity of each immunoprecipitated band to its input.

The IDO1 promoter region was shown to contain three partial RelB/p52 binding sites (AGGAGACACA, GGGAGACAGA, and AGGAGAAAGA), with a consensus noncanonical binding sequence PuGGAGApyTTPu located close to position -2000 **(Fig 4A)**, [21,28,29]. To demonstrate direct binding of RelB/p52 to the IDO1 promoter, we performed a ChIP analysis experiment using RelB binding to pull-down the IDO1 promoter **(Fig 4B)**. Immunoprecipitation of RelB induced by CTB-INS showed binding to all three IDO1 non-canonical binding sequences with increased binding at the GGGAGACAGA promoter sequence **(Fig 4B).**

### Vaccine stimulation of the TNFR signaling pathway induces IDO1 biosynthesis in human DCs

**Fig 5:**
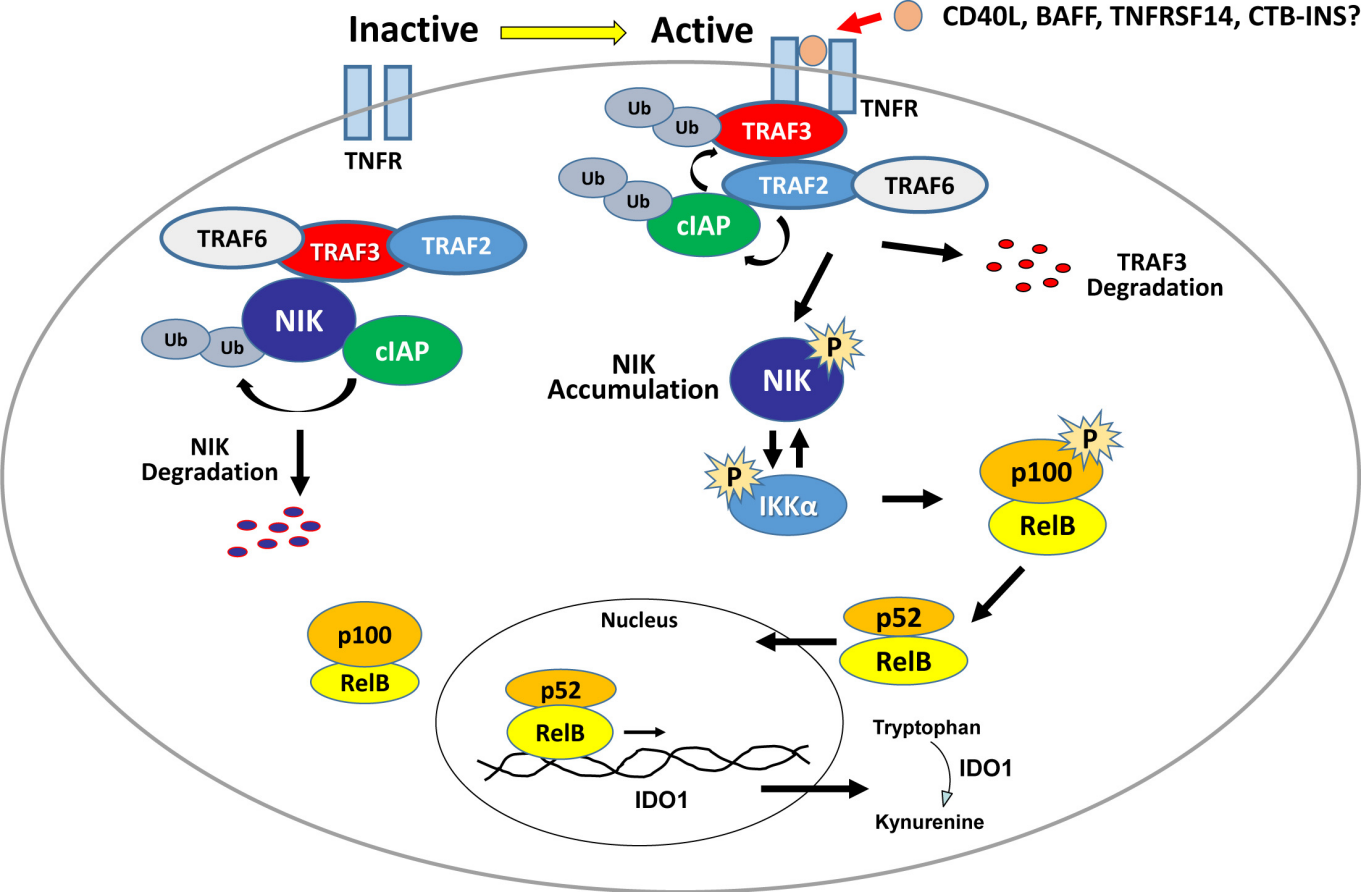
Activation of the non-canonical NF-κB pathway. In the basal inactive state (left), the TRAF-cIAP complex catalyzes ubiquitination of NIK, leading to constitutive NIK degradation in the proteasome leaving p100-containing RelB complexes isolated in the cytoplasm. During activation (right), the TRAF-cIAP complex is recruited to the CD40 receptor. Upon ligand binding, TRAF2-mediated, ubiquitination of cIAP1/2 switches its ubiquitin ligase activity from NIK to TRAF3. The resultant TRAF3 degradation destabilizes the TRAF-cIAP complex allowing accumulation of newly synthesized NIK. Phosphorylated NIK then transfers a phosphate to IKKα. Now activated, IKKα phosphorylates p100 leading to its partial proteosomal degradation releasing p52: RelB heterodimers that translocate into the nucleus. Further, activated IKKα phosphorylates NIK, destabilizing it thereby limiting downstream activation events.

Control of NIK post-translational stability may be essential for non-canonical NF-κB signaling modulation. Therefore, the control of NIK stability is one of the prime questions for understanding regulation of the non-canonical NF-κB signaling pathway. Mounting evidence suggests that TNF receptor-associated factors, TRAF2, TRAF3 and TRAF 6, are critical molecules involved in negative regulation of NIK activity [30–32]. **(Fig 5)** Pharmacological inactivation of these proteins or their deletion also allows for basal NIK accumulation in the absence of ligand [15,17,19,24,25,33,34].

**Fig 6:**
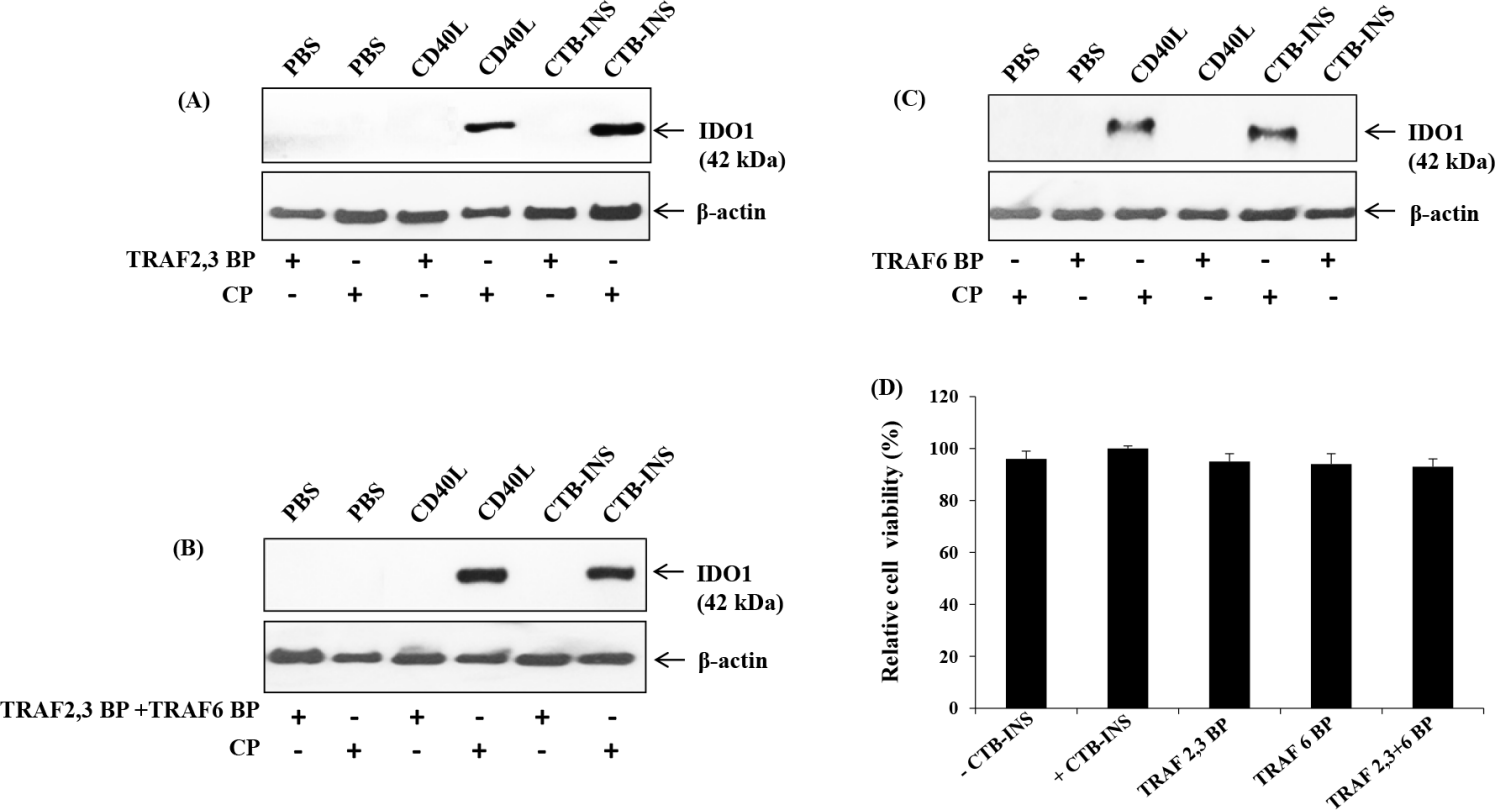
The TNFR-TRAF pathway is required for CTB-INS vaccine induction of IDO1 protein biosynthesis. In Panels **(A,B,C)** monocyte-derived DCs were inoculated with blocking peptides containing the amino acid sequence of TRAF2, 3 or TRAF6 binding sites of CD40 linked to the TAT_47–57_ membrane transport peptide. After blocking peptide binding, the DCs were stimulated with 500ng/ml of CD40L (CD154), Immunex, Seattle, WA), LPS (1μg/ml) and CTB-INS (10μg/ml). Both CD40-TRAF2, 3 and CD40-TRAF6 blocking peptides impaired upregulation of IDO1 in response to CD154, LPS and CTB-INS. Each image is representative of two independent experiments. **(D)** Relative cell viability of cells treated with CTB-INS in combination with TRAF 6BP and TRAF 2, 3 BP.

The engagement of CD40 by CD40L promotes clustering of CD40 inducing the recruitment of adapter proteins known as TNFR-associated factors (TRAFs) to the cytoplasmic domain of CD40 [33]. Previous reports showed that cell permeable peptides that include the TRAF2, 3 or TRAF6 binding site to CD40 are able to block the CD40-TRAF signaling pathway [24,32,33,35–38]. Inhibition of the TNFR pathway permits examination of its role in induction of IDO1 in vaccinated dendritic cells. Therefore, monocyte-derived DCs were incubated with peptides containing the amino acid sequence of the TRAF2, 3 and the TRAF6 binding sites to CD40. The DCs were then stimulated with CD40 ligand (CD154), and CTB-INS. The CD40-TRAF2, 3 and CD40-TRAF6 blocking peptides were shown to impair upregulation of IDO1 in response to CD154, and CTB-INS treatment. The most impairment of IDO upregulation was detected when both TRAF 2, 3 and TRAF 6 inhibitors were used in combination. **(Fig 6)**

### CTB-INS as a ligand for members of the TNFR Superfamily

**Fig 7:**
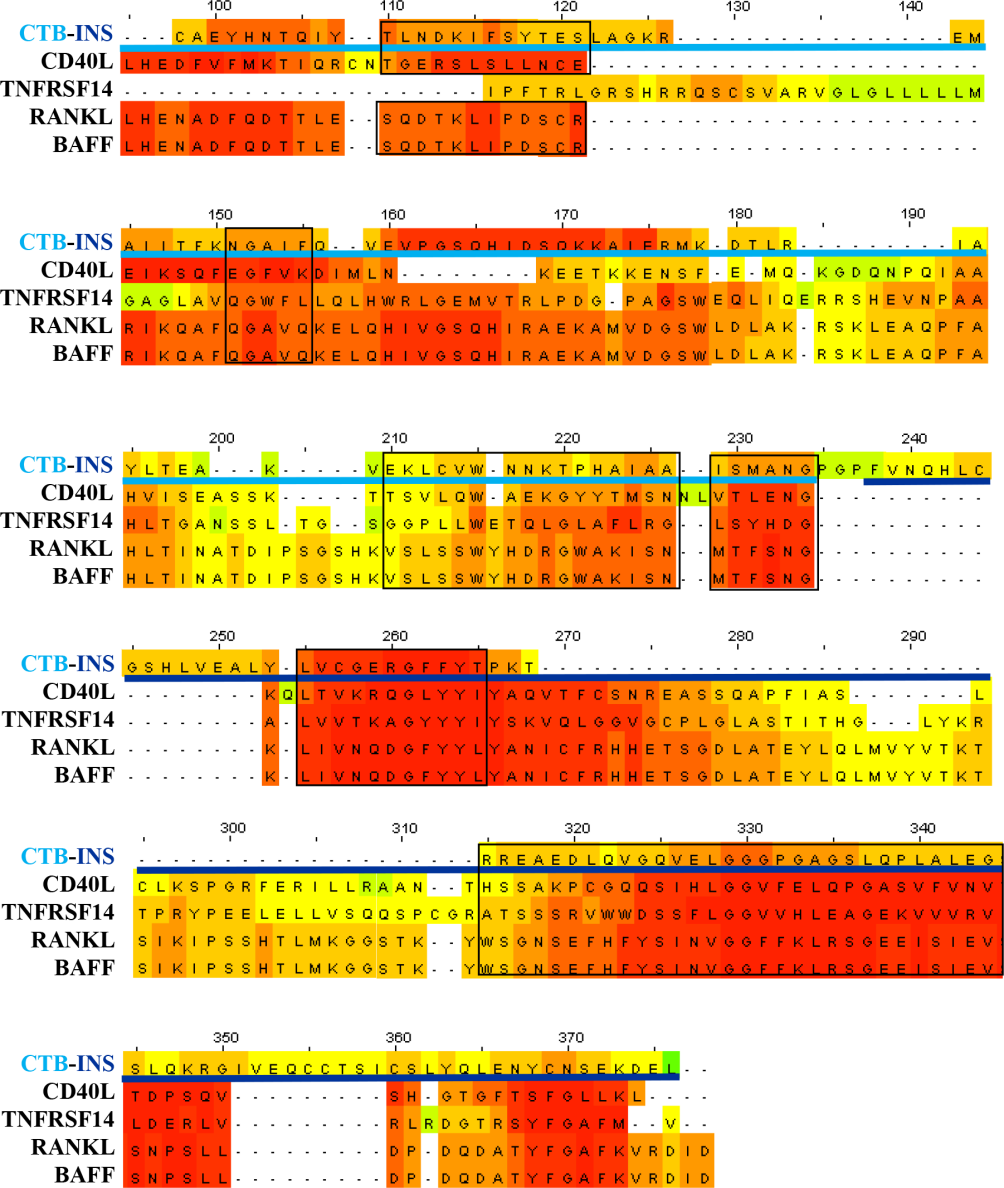
Comparison of the amino acid sequence of CTB-INS with ligands of the Tumor Necrosis Factor Receptor (TNFR) superfamily (CD40L, TNF14, RANKL and BAFF). The light-blue horizontal line corresponds to the amino acid sequence of CTB and the dark-blue horizontal line denotes the amino acid sequence of proinsulin. The black boxes highlight areas of greater functional binding homology among TNFR family members, in which the red highlighted areas signify greater levels of amino acid homology and the green areas, low to no detectable homology.

Members of the Tumor Necrosis Factor (TNF) receptor family have been shown to stimulate DC maturation or modulate peripheral tolerance in autoimmunity by upregulation of IDO1 [14,22,39]. Both CTB and LTB enterotoxin protein binding subunits were shown to stimulate antigen presenting cell CD40 surface expression and DCs were found to upregulate IDO1 mediated immune suppression through activation of the NF-κB non-canonical signaling pathway [14]. Protein functional homology analysis (PROPSEARCH^TM^) identified the probability of functional homology between CTB-INS and the TNF subfamily of ligands to be >87%, (unpublished data). Based on the data in **(Fig 6)** we hypothesize that CTB-INS interacts with TNF receptors to stimulate IDO1 synthesis in vaccinated DCs. To test this hypothesis, we aligned CTB-INS protein amino acid sequence with the following tumor necrosis factor (TNF) superfamily member ligands: CD40L (Accession: NP_000065.1), TNFR14L (Accession: NP_003798.2), RANKL (Accession: NP_003692.1), and BAFF (Accession: NP_006564.1) using the T-Coffee server. We observed that the CTB-INS vaccine shares significant levels of amino sequence homology with ligands of the TNFR superfamily **(Fig 7).**

## Discussion

The mechanism of CTB-INS induction IDO1 biosynthesis was shown to be dependent on the NF-κB signaling pathway [13]. However, the relative contributions of canonical and non-canonical branches of this pathway to IDO1 up regulation remain unknown. Earlier work by Tas and his colleagues showed that CD40L was responsible for stimulation of IDO1 via the non-canonical pathway [14], suggesting this pathway could play a significant role in CTB-INS induction of IDO1 in human DCs. Based on NIK-dependent activation experiments, our data suggest CTB-INS induces IDO1 in human monocyte-derived DCs via the non-canonical NF-κB pathway. In addition, ChIP analysis experiments showed that NF-κB RelB-p52 dimers bound to defined consensus sequences within the *IDO1* promoter *in vivo*, suggesting the non-canonical signaling pathway is active in vaccine induction of IDO1 in human DCs. Blocking TRAF adaptor molecule functions was shown to inhibit IDO1 biosynthesis in vaccinated DCs suggesting upregulation of IDO may occur through TNF receptor family stimulation of the NF-κB non-canonical signal transduction pathway [31]. In the TNF-activated signal transduction pathway, NIK is known to interact with TRAF2, and TRAF3 leading to non-canonical NF-κB activation [32].

Induction of IDO1 depends on fusion of CTB to proinsulin [13], suggesting that the vaccine may bind as a ligand to receptors responsible for NF-κB non-canonical pathway activation of IDO1 expression. Several ligands of the Tumor Necrosis Family Receptor (TNFR) superfamily were shown to activate the non-canonical NF-κB pathway [14,40–44]. For suggestive evidence of a link between CTB-INS with TNFR, we compared amino acid sequences of CTB-INS with those of four ligands of the TNFR family to assess any type of functional homology. We found that several areas where the vaccine could act as a TNFR ligand, represented by areas of greater amino acid homology **(Fig 7).**

The association between the ligand and its potential receptor suggests that CTB-INS receptor binding may involve specificity of the autoantigen for its receptor rather than existing as a general mechanism for binding all CTB-autoantigen conjugates. Our experimental data suggests that CTB-INS induces non-canonical NF-κB signaling which is driven by TRAFs as TNFR signal mediators. Although, there is evidence that CTB-INS elicits immunosuppressive effects through TNFRs, further experiments are needed to determine the probability of CTB-INS binding to individual members of the TNFR family.

The B-cell activating factor (BAFF) predominantly expressed in B cells, differs from many other TNFR superfamily members in that it generally activates the non-canonical NF-κB signaling pathway with only weak induction of canonical NF-κB pathway signaling [41,45]. This unique feature of the BAFF receptor (BAFFR) is due primarily to its possession of an atypical TRAF-binding sequence, which interacts with TRAF3 but not with TRAF2 [41]. The BAFFR-mediated induction of p100 processing to p52 contributes to the survival of transitional and mature B cells, likely through induction of anti-apoptotic genes like *bcl-2* and *bcl-x* [41,45].

The CD40 molecule is a TNFR member expressed on a variety of cell types, including B cells, dendritic cells, monocytes, endothelial epithelial cells, and neurons [39,46]. Activated T cells primarily express the ligand of CD40, alternatively referred to as CD40L or (CD154). In the immune system, a major function of CD40 signaling is to regulate B-cell activation and differentiation events, including proliferation and survival of activated B cells, germinal center formation, and antibody isotype switching. Another major function of CD40 is to mediate dendritic cell maturation and antigen presentation. Unlike BAFFR, CD40 elicits strong signals that target both the canonical and non-canonical NF-κB pathways [14,39]. Upon ligation by CD40L, CD40 interacts via two different TRAF-binding motifs that include TRAF1, 2, 3, 5, and 6, and this interaction leads to proteolysis of both TRAF2 and TRAF3 [43,47]. As indicated above, the degradation of TRAF2 and TRAF3 represent an important step in the activation of the non-canonical NF-κB signaling pathway [15,16,34].

The herpesvirus entry mediator (HVEM) or tumor necrosis factor receptor superfamily member 14 (TNFRSF-14) is a protein originally known as herpesvirus entry mediator A (HveA). Both HveB and HveC are structurally unrelated proteins of the immunoglobulin superfamily [48]. HvA is also known as Cluster of Differentiation CD270 [40]. Moreover it is also referred to as ATAR (another TRAF-associated receptor). Interactions between TNFRSF-14 and TRAF2 were shown to activate the non-canonical NF-κB signaling pathway [40].

The Receptor Activator of Nuclear Factor κ B (RANK) is best known for its role in osteoclastogenesis [15,44]. However, it also regulates important immune functions that include dendritic cell survival and lymphoid organogenesis [42]. RANK is expressed on osteoclast precursors, dendritic cells, and activated B cells, and in general, RANK signaling was shown to promote cell survival and differentiation. Analogous with CD40, the cytoplasmic domain of RANK was shown to bind TRAF1, 2, 3, 5, and 6 and mediates activation of both canonical and non-canonical NF-κB signaling pathways. Genetic evidence suggests an essential role for RANK-stimulated activation of non-canonical NF-κB activation during osteoclastogenesis and bone metabolism [44]. The non-canonical NF-κB has been closely linked to immune suppression [13,49]. Several ligands such as Glucocorticoid-induced tumor necrosis factor receptor (GITR) on T cells and its natural ligand, GITRL, on accessory cells contribute to the control of immune homeostasis. Grohmann et al. showed that reverse signaling through GITRL after engagement by soluble GITR initiates the immunoregulatory pathway of tryptophan catabolism in mouse plasmacytoid dendritic cells, by means of noncanonical NF-κB–dependent induction of IDO1 [50]. Additionally, CpG-rich oligodeoxynucleotides activate the immune system, leading to innate and adaptive immune responses that have been shown to promote tolerogenic responses in mouse plasmacytoid dendritic cells in vivo and in an *in vitro* human DC model. Unveiling a previously undescribed role for TRIF and TRAF6 proteins in Toll-like receptor 9 (TLR9) signaling, it was demonstrated that physical association of TLR9, TRIF and TRAF6 leads to activation of non-canonical NF-κB signaling and the induction of IRF3- and TGF-β-dependent immune-suppressive tryptophan catabolism [51].

Understanding the link between vaccine activation of TNF receptor family members and the activation of non-canonical NF-κB signaling is an important step in elucidation of the mechanism underlying chimeric vaccine induction of immunological tolerance in dendritic cells. Understanding the mechanism of chimeric vaccine modulation of IDO1 induction and suppression of in human dendritic cell activation will facilitate development of chimeric vaccine strategies for effective and safe therapy for type 1 diabetes and a wide range of tissue specific autoimmune diseases.
